# Identification of growing tuberculosis incidence clusters in a region with a decrease in tuberculosis prevalence in Moscow (2000-2019)

**DOI:** 10.7189/jogh.13.04052

**Published:** 2023-05-26

**Authors:** Alexei A Romanyukha, Arseny S Karkach, Sergey E Borisov, Evgeny M Belilovsky, Tatiana E Sannikova

**Affiliations:** 1 Marchuk Institute of Numerical Mathematics, Russian Academy of Sciences, Moscow, Russia; 2 Moscow State University, Moscow, Russia; 3 Moscow Research and Clinical Center for Tuberculosis Control, Moscow Department of Public Health, Moscow, Russia

## Abstract

**Background:**

The control of tuberculosis (TB) may benefit from a prospective identification of areas where the incidence may increase in addition to the traditionally identified foci of high incidence. We aimed to identify residential areas with growing tuberculosis incidence rates and assess their significance and stability.

**Methods:**

We analysed the changes in TB incidence rates using case data georeferenced with spatial granularity to apartment buildings in the territory of Moscow from 2000 to 2019. We identified sparsely distributed areas with significant increases in the incidence rate inside residential areas. We tested the stability of found growth areas to case underreporting via stochastic modelling.

**Results:**

For 21 350 cases with smear- or culture-positive pulmonary TB among residents from 2000 to 2019, we identified 52 small-scale clusters of growing incidence rate responsible for 1% of all registered cases. We tested clusters of disease growth for underreporting and found them to be relatively unstable to resampling with case drop-out, but their spatial displacement was small. Territories with a stable increase in TB incidence rate were identified and compared to the rest of the city, which is characterised by a significant decrease in incidence.

**Conclusions:**

Identified areas with a tendency for an increase in the TB incidence rate may be important targets for disease control services.

Tuberculosis (TB) is one of the leading causes of mortality globally, particularly in low- and middle-income countries. Moscow, the capital of Russia, has witnessed a steady decrease in TB prevalence for the last 14 years, yet there are still areas within the city where TB incidence is increasing, making control and management a significant health challenge. The control of TB requires significant resources, including funding, medical personnel, and equipment. Given their finiteness, these resources must be allocated effectively and efficiently, making it crucial to identify areas with high TB prevalence. We aimed to identify and characterise the clusters of TB incidence growth in Moscow over a twenty-year period, utilising spatial analysis techniques. 

We studied the epidemiology of pulmonary TB among residents of Moscow, i.e. people who have been registered in the city for the past 10 years. The city’s resident population is 12 million (estimated registered population living within the Moscow Ring Road, as the effective number of people may be higher due to commuting); 98% lives in the part of the city limited by the Moscow Ring Road. This part of the city is divided into 107 municipalities, whose residents differ in social, economic, and demographic characteristics, as well as up to three to four times in incidence of TB.

From 2009 to 2020, Moscow experienced a decrease in the incidence rate of pulmonary TB among residents from 24 to 6.5 per 100 000 population, and the incidence rate of active laboratory-confirmed pulmonary tuberculosis ((MTB+) [1]) dropped from 12 to 2.7 cases per 100 000 population. About 70% of cases were detected during preventive examinations of at-risk groups. Despite the efforts, 2245 cases of pulmonary TB were detected in 2020, of which 952 were among residents, highlighting a significant infection importation and risk for the emergence of locations with an increased risk of infection. Immigrants settle in the city mostly diffusely, except for few areas with expensive real estate (<5% of the territory). Housing is generally rented for a year or more. Published works [2] note that compact enclaves with migrants are concentrated outside the Moscow Ring Road in areas of intensive construction, while the localisation of migrants inside it is diffuse and associated with renting housing near place of employment. In such circumstances, local areas with an increased risk of infection can emerge; locating them is important for TB prevention and control services.

The development of methods for detecting spots with an increasing incidence rate in the city, despite a decreasing average incidence rate, will allow us to identify emerging territories with an increased risk of infection. Through this study, we aimed to search for residential areas with a significant increase in morbidity and study the mechanisms that determine their emergence. Once identified, such spots are a priority target for anti-TB activities. To solve this problem, we extended the approach proposed in another study [3].

We searched for reviews on the epidemiology of pulmonary TB published, from 2017 to 2022. The review by Shaweno et al. [4] provided a valuable reference for approaches to the spatial analysis of TB epidemiology. Few studies interpreted the growth of TB incidence clusters as an increase in the size of those clusters or the number of cases in them over time. The following studies define a cluster as a small group (two or more) of genetically identical cases and cluster growth as an increase in cases of a given genotype in a given place.

Driver et al. [5] examined factors influencing TB cluster growth, investigating clusters of two or more genotyped and culture-confirmed *Mycobacterium tuberculosis* cases with insertion sequence 6110 (IS6110) restriction fragment length polymorphism and spoligotype patterns identical to those of another study case. They examined the effect of the combined characteristics of infectiousness of the first two cases in a cluster on the rate of cluster growth. Patients’ infectiousness was not associated with the rate of cluster growth among historical strain clusters. Among recent strain clusters, the infectiousness of both initial cases was associated with a higher rate of cluster growth compared to clusters in which neither initial case was infectious after adjustment for male sex. The rate of genotype cluster growth should be monitored, regardless of how long the strain has been present in the community. However, the infectiousness in the first two cases may be useful to prioritise genotype cluster investigations.

Althomson et al. [6] used TB patient characteristics, genotyping data, and geolocation to predict which small clusters (more than three cases) were most likely to become outbreaks of at least six TB cases; 11.0% of the studied clusters grew into outbreaks. The discovered factors of outbreaks were homelessness, excessive alcohol, or illicit drug use. The study suggests that routinely reported data may identify small clusters that are likely to become outbreaks and, therefore, are candidates for intensified contact investigations.

Outbreaks among incident clusters of TB were defined as clusters in which the initial case was preceded by at least 24 months of no genotype-matched cases within a geographic area.

Althomson et al. [7] studied the differences between new cases due to reactivation and transmission. They proposed a method to detect possible outbreaks among endemic TB clusters. This method cannot be applied to endemic clusters (without genotyping) because the initial case cannot be determined. These endemic clusters may be a combination of cases that are the consequence of reactivation of TB in persons who were previously infected and recent transmission of TB. The method searches for instances of excessive, unexpected cluster growth above a background rate.

Rengganis et al. [8] investigated the dynamics and characteristics of social determinants of spatiotemporal TB clusters and identified the characteristics of population density and percentage of poverty of the clusters. Geographical coordinates of the TB patients and secondary data, consisting of population density and the percentage of poverty, were used for the region under investigation. They analysed the data with a space-time permutation model using the SaTScan software [9]. The authors found spatiotemporal dynamics of TB clusters, including the number of clusters with significant dynamics, TB cases within the clusters, and the placement and sizes of the clusters, all of which had similar social determinant characteristics: medium-high population density and low-medium percentage of poverty.

Althomsons et al. [10] studied predictors of increased incidence in clusters based on genotyped culture-positive TB data. They defined clusters as three or more TB cases with a matching genotype reported in the same US county or equivalent jurisdiction. The authors aggregated counts for each cluster according to calendar year quarters to identify any unexpected growth by fitting each cluster by a negative binomial hurdle (NB) model, which is a class of statistical models where a random variable is modelled using two parts: the first is the probability of attaining the value 0, and the second is the probability of the nonzero values. The use of hurdle models is often motivated by an excess of zeros in the data that is not sufficiently accounted for in more standard statistical models. They were introduced by Cragg [11], where the nonzero values of x were modelled using a normal model and the zeros using a probit model. An eight-quarter baseline represents expected cases before the detection of unexpected growth. For each cluster, the NBH model sets the threshold for unexpected growth during the next quarter as the 95th percentile of the baseline.

## METHODS

**Table 1** lists the most important characteristics of TB case data.

**Table 1:** Parameters and quantities of tuberculosis cases and population*

Parameter	n
Moscow population under consideration*	12 000 000
Cases of MTB+ (TB register, 2000-2019)	21 350
Unique addresses of MTB+ cases up to apartment building	13 741
Clusters of significant incidence rate increase†	52
Cases in clusters of significant incidence rate increase†	219
Average cases per cluster of significant incidence rate growth†	7.5/4.2
Stable clusters of significant incidence rate increase‡	31
Total cases in stable clusters of significant incidence rate increase‡	139
Average cases per cluster of stable, significant incidence rate growth‡	5.1

MTB+ – active laboratory-confirmed pulmonary tuberculosis, TB – tuberculosis

*Residents living inside the Ring Road, data for 2000-2019.

†For 100% of cases, without and with overlap. Many clusters are significantly overlapping, and some cases belong to several clusters.

‡Clusters stable to case dropout were defined as appearing in at least 50% of the maximum number of occurrences in 1000 sampling runs with 5% case dropout.

### Data

We used the individual case data from the continuously updated TB register of Moscow patients [12] and selected cases from the area within the Ring Road (the main part of the contemporary city) only, justified by the better completeness of casa data in these municipalities. There were 21 350 first cases of infectious smear or MTB+ registered in 2000-2019 among the resident population of this territory. The individual case data were registered at 13 741 residential addresses that were geocoded down to residential buildings, usually apartment blocks, using the Yandex geocoding web service [13].

### Dynamics of the local incidence rates

**Figure 1:**
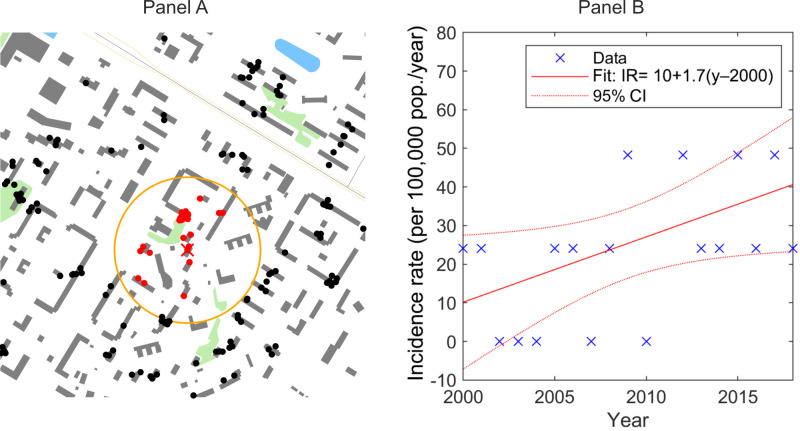
Estimation of the local rate of change in incidence rate. **Panel A.** Location (orange) – a 200 m circle around the position of a case (red cross), other cases reported within this location (red dots), and cases reported outside this location (black dots). **Panel B.** Estimation of the local rate of change of the incidence rate. Cases detected at a location by year (“x”), trend of the incidence rate estimated by a linear regression (solid line), and the 95% confidence intervals (dotted lines). Incidence rate values are “quantised” due to the discrete nature of case counts per location per year (0, 1, 2 etc.).

To estimate the trends of local incidence rates, we used the “screening circles” of radius *R* = 200m centred at geographical coordinates (usually apartment buildings) of every MBT+ case reported [3], to which we refer to as “locations” (**Figure 1**, Panel A). By sampling the cases within them, we calculated the case count for every year and location. Assuming the average population density in the studied area is 33 000 persons/km^2^, we estimated the local incidence rates for each year for all case locations.

We obtained rates of change (regression slope) for the local incidence rates and their significance for all locations (**Figure 1**, Panel B) by fitting the local incidence rates with a linear regression model [14]. This allowed us to discover locations with a significant growth or decline in the MTB+ incidence rate, i.e. the “clusters of incidence rate growth”.

### Stability of clusters of incidence rate growth

Due to the rare occurrence of MTB+ cases, only 7.5 cases are observed per cluster annually (**Table 1**), and the “local” incidence rate and its growth rate are prone to random variation.

**Figure 3:**
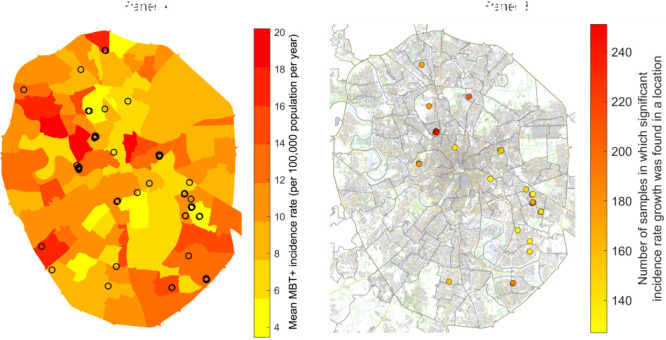
Clusters with significant MTB+ incidence rates growth. **Panel A.** Choropleth map of average MTB+ incidence rates in municipalities (years 2000-2019) and 52 clusters of significant MTB+ incidence rate growth. **Panel B.** 31 stable clusters of MTB+ incidence rate growth that are most resistant to missing cases.

We applied the following sampling technique to study the stability of the local incidence rate. Of all MTB+ cases, we randomly dropped 5% and recalculated the local incidence rates, their growth, and significance using the remaining 95% of cases. Such sampling was repeated 1000 times. For each cluster, we calculated the number of times the significant growth of incidence rate was observed. A maximum of 251 sampling experiments a significant growth was observed in the same cluster (i.e. same location). Growth in other clusters was observed less frequently. We selected the clusters in which significant growth was observed 50% of the times or more (125 times or more) among the sampling experiments, as compared to the most frequently observed cluster. We found 31 of such clusters, designating them as the stable clusters of significant MTB+ incidence rate growth (**Figure 3**).

### Effective reproduction number

**Figure 4:**
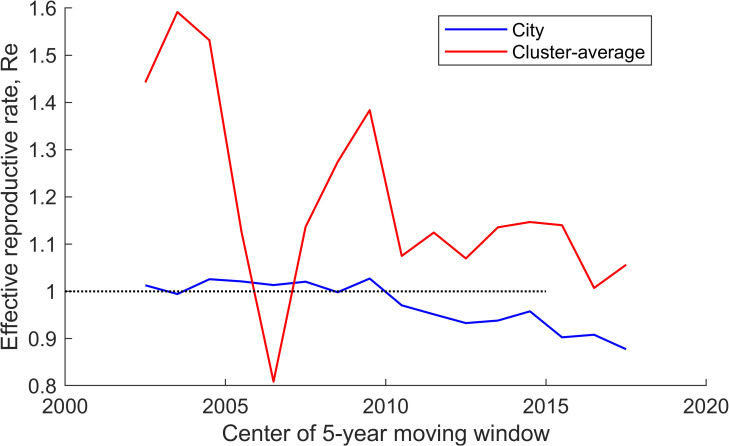
Effective reproductive number (*R_e_*) of MTB+ calculated for Moscow (blue) and 52 clusters of significant incidence growth in five-year windows (red).

The effective reproductive number (*R_e_*) is the expected number of new infections caused by an infectious individual in a population where some individuals may no longer be susceptible. It is an epidemic parameter used to assess whether an epidemic is growing, shrinking, or holding steady. *R_e_* estimates can be used as an indicator of epidemic growth, to assess the effectiveness of interventions, and how changes in policy, population immunity, and other factors have affected transmission at specific points in time [15,16]. There are different methods of estimating it, accounting for delays between infection and onset of the disease. We used the approach described and proposed elsewhere [17,18]. According to Wallinga and Lipsitch’s [19] relatively simple approach based on the generation interval method, *R_e_* can be approximated using the following equation:

*R_e_*  = 1 + *rT*

where *r* is the rate of exponential growth (or per capita increase in the number of new cases per year) and *T* is the mean generation interval (or the period between infection of an index case and detection of a secondary case generated from the index case). The value of *r* can be obtained by fitting the number of new cases (from the time series data of new cases at population level) and assuming an exponential curve. We fitted data for the city-average incidence rate and the average annual incidence rate in 52 clusters of significant incidence rate growth. Since the epidemiological situation was not stable over the 20-year period, and to get a temporal perspective of *R_e_*, we used data in a five-year sliding window (2000-2004, 2001-2005, etc.) and calculated *R*_e_ for the city and clusters for each temporal interval. The *T* was assumed to be equal to one year (**Figure 4**).

## RESULTS

**Figure 5:**
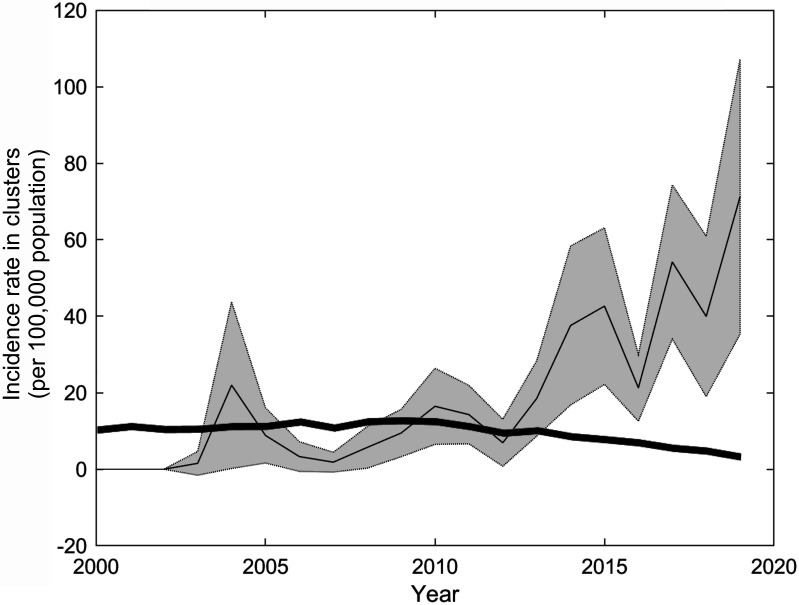
Incidence rate in 52 clusters of significant MTB+ incidence rate increase (thin solid line) with 95% confidence intervals (grey area). Thick line – average rate of MTB+ incidence.

We obtained a method for detecting clusters of a significant incidence rate increase and found clusters in which the incidence rate of MTB+ increased from 0 to 60 cases per 100 000 population in 2000-2019 (**Figure 5**).

**Figure 2:**
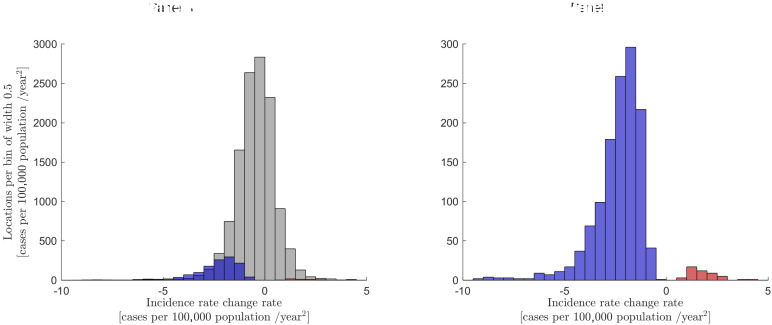
Distribution of rates of change in incidence rates for cases in locations. **Panel A.** Data for 21 350 cases: grey - no significant changes (19 096 cases), blue - significant decrease (2179 cases), red - significant increase (75 cases). **Panel B.** Cases with significant incidence rate trends only. Horizontal axis: the rate of incidence rate change in locations from 2000 to 2019.

**Figure 2** shows the distribution of rates of change in incidence rate for cases in locations estimated for years 2000-2019. Among the sampling circles drawn around each of 21 350 cases with all the neighbouring MBT+ cases of 2000-2019 registered inside them, 19 096 (89.4%) had no significant change, 2179 (10.2%) had a significant decrease, and 75 circles (0.4%) had a significant increase in the MTB+ incidence rate. The right tail of the distribution represents 52 locations, each of which consists of five to six apartment buildings and 5000-7000 residents on average. From 2000 to 2010, the incidence of TB among residents of these 52 locations increased from almost zero to 60 cases per 100 000 population (**Figure 5**). These results show that about 150 000 residents living in areas of increasing incidence have a high risk of TB infection.

**Figure 4** shows the dynamics of *R_e_* in clusters of growing incidence rate, which were unstable. They were at 1.3-1.6 in the first five years, after which they decreased to 1.1-1.2. The reason for the sharp decline in *R_e_* in 2005-2007 is an artefact of low case counts. In 2009, the value of *R_e_* returned to the trajectory of a monotonic decrease to 1. The effective reproductive number for the average MTB+ incidence rate in Moscow from 2000 to 2010 was 1.02-1.03, after which it declined monotonically to 0.9. A comparison of the characteristics of TB patients identified in clusters and on average in the city showed that patients in clusters were more likely to experience relapses and decays (**Table 2**).

**Table 2:** Comparison of epidemiological and demographic indicators in 52 clusters of significant MTB+ incidence rate increase (219 patients) and in the city (77 160 patients)

Parameter	City-wide for residents within the Ring Road who have a history of TB	For clusters with a significant incidence rate growth
Average age	44.7 (SD = 0.1)	42.9 (SD = 1.1)
Proportion of men	73.0% (SD = 0.3%)	68.5% (SD = 3.1%)
Proportion with lung cavities	21 710 of 77 160 patients (28.1% (SD = 0.2%))	118 of 219 patients (53.9 (SD = 3.4%))
Proportion with relapses	5816 of 77 160 (7.5% (SD = 0.1%))	23 of 219 selected patients experienced a relapse (at some point in their disease history) (10.5% (SD = 2.1%))

TB – tuberculosis, SD – standard deviation

**Table 2** shows the epidemiological and demographic characteristics of patients with MTB+ detected in 52 clusters of significant growth, in comparison with the average characteristics of all TB patients diagnosed in 2000-2019. For the cases in the growth clusters, there was a significantly lower proportion of men (68.5% vs 73%), a slightly lower age of newly diagnosed cases of MTB+ (42.9% vs 44.7%), a significantly higher proportion of patients with lung cavity (53.9% vs 28.1%), and a significantly higher proportion of relapses (10.5% vs 7.5%).

The number of locations with a significant decrease in incidence rate is 29 times greater than the number of locations with an increase. This corresponds to the fact that the city-average incidence rate is declining. About 1% (approximately 150 000) of the population lives in locations with a significant increase in the incidence rate. Maps of clusters with a significant increase in incidence rate and with a significant and stable increase in incidence rate are shown in **Figure 3**, panels A and B.

**Figure 3**, Panel A shows that clusters with a significant increase in incidence are located in the central and southeastern parts, and a significant decrease in incidence is in the northern, northeastern, and southern parts of the city where the highest incidence was detected. Note that the central part of the city is relatively prosperous, and the southeastern parts are much less well-off. Significant decreases in the incidence rate were found in the northern, northeastern, and southern parts of the city, where the highest incidence rates were detected. **Figure 5** shows the dynamics of the average incidence in clusters of significant growth.

## DISCUSSION

We previously identified clusters of high incidence rate of pulmonary TB on the periphery of Moscow [3]. Here, we aimed to investigate how such clusters emerge and to detect clusters of growing incidence rate before they reach high levels, i.e. to identify the emerging foci of growing incidence and take preventive measures. Achieving this goal was complicated by a small number of cases of MTB+, which led to the instability of the identified clusters.

Clusters of the growing TB incidence rate were small in size (R = 200 m), located inside residential quarters, and did not have features in their vicinity that could increase the probability of infection/activation of TB (markets, train stations, TB clinics, etc).

The position of growth clusters is different from the position of high incidence clusters. **Figure 3**, Panel A shows that the emergence of TB growth clusters does not depend on the incidence in the municipality, which varies from 4 to 18 cases per 100 000 population. **Figure 5** shows that the initial frequency of MTB+ cases in clusters of increasing incidence rate is close to zero.

The difference in epidemiological and demographic indicators in clusters of significantly increasing MTB+ incidence rate and in the city (**Table 2**) presumably indicates an increase in the proportion of the socially maladjusted population in clusters. According to epidemiologists, such populations may be permanent residents of Moscow, living on a low pension, and commonly exhibit bad habits (smoking, drinking alcohol). Geographically, clusters were located in low-comfort buildings (built in the 1960s and earlier) with ageing populations. The socially maladjusted exhibit decreased compliance with medical interventions and inattention to their health. An increase in the proportion of women among patients indicates a likely increase in the frequency of intrafamilial transmission of infection.

Regarding the incidence of TB growing in 52 locations and decreasing or remaining unchanged in 98.5% of the city, it should be noted that there are usually five to six apartment buildings per location, all of which belong to residential areas. Comparison of the characteristics of patients identified in clusters with the average characteristics of patients for the whole city did not reveal significant differences, except for a significant increase in the frequency of decays and relapses in patients from the clusters. These deviations indicate late detection and low adherence to treatment in TB patients in areas of increasing incidence. Free medical care, including diagnostics and hospital treatment, is available to everyone, and the Moscow anti-TB service system, experiencing the decrease in the TB incidence, was not overloaded and had sufficient funding. Clusters were located in municipalities with different levels of morbidity and attached to different territorial divisions of the TB service. Therefore, late detection of infection was not associated with monitoring deficiencies. The reasons for the behaviour of residents of clusters of growing MTB+ incidence rate and the possible role of importation in the appearance of clusters need further research.

## CONCLUSIONS

We propose a method for assessing the spatial heterogeneity of the dynamics of morbidity and identifying clusters of increasing morbidity. Identified areas with a tendency for an increase in the TB incidence rate may be important targets for disease control services. We demonstrated that our approach can identify high-risk TB clusters with a tendency for an increase in the TB incidence rate, which could aid policymakers in targeting resources to specific regions for prevention and control interventions. Our findings underscore the importance of employing spatial analytical methods in understanding disease transmission dynamics and controlling TB in urban setting.
